# Unraveling the Hippo pathway: YAP/TAZ as central players in cancer metastasis and drug resistance

**DOI:** 10.17179/excli2025-8351

**Published:** 2025-06-06

**Authors:** Nehmat Ghaboura

**Affiliations:** 1 Department of Pharmacy Practice, Pharmacy Program, Batterjee Medical College, Jeddah 21442, Saudi Arabia

## Abstract

In regulating cellular plasticity, epithelial to mesenchymal transition (EMT), and tumor progression across a broad range of cancer types, the Hippo signaling pathway depends on YAP (Yes-associated protein) and TAZ (transcriptional coactivator with PDZ binding motif) as core effectors. This pathway can become dysregulated, disrupting tissue homeostasis and promoting oncogenic processes such as metastasis, immune evasion, and therapy resistance. This review explores the multifaceted roles of YAP/TAZ in lung, breast, ovarian, liver, and renal cancers, detailing their interactions with key signaling pathways such as TGF-β, Wnt, and PI3K/AKT and their modulation by mechanical cues like extracellular matrix stiffness and fluid shear stress. Potential YAP/TAZ mediated therapy resistance in EGFR TKI-resistant lung cancer and platinum-resistant ovarian cancer and the impact this has on tumor metabolism as a result of YAP/TAZ controlling tumor mesenchymal stem cells in the hypoxic environment of hepatocellular carcinoma is highlighted. Additionally, we discuss their role in maintaining cancer stem cell traits, creating an immunosuppressive tumor microenvironment, and driving chemoresistance in breast and renal cancers. Small molecule inhibitors, natural compounds (luteolin, apigenin, honokiol), and novel agents (nanoparticles of zinc oxide) are discussed as promising routes for disrupting YAP/TAZ. The review underscores the complexity of YAP/TAZ signaling and the need for patient stratification based on their expression levels to optimize targeted therapies.

See also the graphical abstract(Fig. 1).

## Introduction

The Hippo signaling pathway, a highly conserved process for organ size control, tissue homeostasis, and cell fate, is highly conserved in the ectoderm (Zhao et al., 2011[179]). These routes were initially detailed in Drosophila melanogaster based on a phenotype of tissue overgrowth reminiscent of a 'hippopotamus' (Pan, 2010[110]). Its core consists of a cascade of kinases, including MST1/2 (homologs of Drosophila Hippo) and LATS1/2, and when broken, this pathway becomes crucial (Meng et al., 2016[93]). Through scaffold proteins SAV1 and MOB1, these kinases phosphorylate and inactivate the TAZ and YAP (Zhao et al., 2020[182]). Under these conditions, phosphorylated YAP/TAZ is retained in the cytoplasm or degraded and cannot localize to the nucleus and become transcribed (Shreberk-Shaked and Oren, 2019[136]). On the other hand, when the pathway is dysregulated, unphosphorylated YAP/TAZ moves to the nucleus. It interacts with TEAD transcription regulators to stimulate genes responsible for the growth of cells, survival, and migration (Mokhtari et al., 2023[98]). The hippo pathway is not just kinase activity-dependent (Yu et al., 2015[168]). Signals to the pathway include ECM stiffness, mechanotransduction, and cell-cell junctions (Di et al., 2023[29]). Upstream regulators, including hormone-regulating G-protein coupled receptors (GPCRs), TGF-β, and WNT signaling, also modulate Hippo activity (Sileo et al., 2022[137]). This dysregulated pathway disrupts the cellular equilibrium, allowing for oncogenic processes of tumor growth, metastatic potential, or therapeutic resistance (Hirschey et al., 2015[45]).

The oncogenic influence of the Hippo pathway is achieved through the TAZ and YAP (Cunningham and Hansen, 2022[23]). These transcriptional coactivators have been shown to function as critical tumorigenic drivers, linking extracellular signals to gene expression programs, promoting cellular proliferation, preventing apoptosis, and enabling migration (Talukdar and Chatterji, 2023[142]). Under normal conditions, their roles are tightly monitored. Still, inappropriate initiation of YAP/TAZ has been detected in several human cancers like lung, breast, ovarian, liver, and renal cancers (Nguyen and Yi, 2019[105]). YAP/TAZ integrates many inputs, including mechanotransduction (e.g., ECM stiffness) (Dupont, 2016[31]). For example, in the tumor microenvironment, increased tissue rigidity induces YAP/TAZ nuclear localization and activation of oncogenic genes (Ortega et al., 2021[109]). The ability of cancer cells to survive and be aggressive under conditions that would otherwise restrict them to survival (Mathew et al., 2007[90]). Like any other oncogenic proteins, YAP/TAZ also couples with well-known oncogenic pathways, i.e., TGFβ, WNT, PI3K/AKT, and magnifies their downstream signaling (Piccolo et al., 2023[115]). However, these interactions further strengthen the oncogenic network, and YAP/TAZ become critical mediators of cancer progression (Zanconato et al., 2016[172]). YAP/TAZ activation has averted therapeutic resistance in several cancers (Kim and Kim, 2017[60]). YAP/TAZ promotes the expression of anti-apoptotic and drug-resistance genes in lung cancer (Liang et al., 2024[70]). Like in mature triple-negative cell panels, YAP/TAZ promotes chemotherapy resistance by preserving cancer stemness and immune evasion in TNBC (Huang et al., 2024[51]). YAP/TAZ sustains stem-like characteristics in tumor cells, reflecting the critical role of YAP/TAZ in tumor development and metastasis (Luo et al., 2023[85]).

YAP/TAZ is also one of the most critical players of EMT, a process that cells undergo, losing their relationships with other cells and assuming a motile and invasive phenotype (Zhang et al., 2021[174]). Metastasis is made possible in part by EMT, whereby cancer cells invade distant tissues (Yao et al., 2011[167]). EMT is regulated by YAP/TAZ by transcriptional modulation of key transcription factors like ZEB1/2, Twist, and Snail (Ichikawa et al., 2022[55]). Through these interactions, they drive the gain of mesenchymal markers like vimentin (VIM) and N cadherin (N-Cad) and the loss of epithelial markers such as E cadherin (E-Cad) (Rubtsova et al., 2022[126]). Analysis of cancer-specific YAP/TAZ signaling effects on tumor aggressiveness and EMT (Luo et al., 2023[85]). YAP/TAZ promotes resistance to anticancer drugs and increases migratory capacity in lung carcinoma (Lo Sardo et al., 2018[79]). YAP/TAZ promotion of stemness and plasticity in breast tumors contributes to cancer evasion of immune responses and adaptation to hostile environments (Guo and Han, 2023[39]). YAP/TAZ contributes to mechanotransduction in liver cancer and enables cancer cells to adapt to increased tissue stiffness, an important hallmark of hepatocellular carcinoma (Lee et al., 2024[65]). Like in ovarian and renal cancers, YAP/TAZ deregulation is a predictor for poor outcomes, indicating involvement in metastatic progression and pharmacotherapy resistance (Luo et al., 2023[84]).

The Hippo-YAP/TAZ pathway maintains vital functions in numerous physiological and pathological conditions since its identification as a cancer pathway. YAP/TAZ signaling dysfunction during cardiovascular disorders induces cardiac hypertrophy, vascular remodeling, and atherosclerosis through its control of endothelial and smooth muscle cell actions (Dai et al., 2024). Neuronal cell death alongside diminished neurogenesis occurs as a result of an abnormally active Hippo pathway in patients with Alzheimer's and Parkinson's diseases (Dong and Jiang, 2024[30]). The embryonic development, tissue regeneration, and organ size regulation are controlled by YAP/TAZ, which also regulates stem cell development and differentiation. The multiple liver cell functions of YAP/TAZ demonstrate their profound biological significance, requiring specific therapeutic approaches to minimize adverse effects during cancer interventions.

This review seeks to understand the dysregulation of the Hippo pathway and the significance of YAP/TAZ in cancer biology. The review synthesizes lung, breast, ovarian, liver, and renal cancer findings, highlighting distinct and overlapping YAP/TAZ dysregulation mechanisms.

## The Hippo Signaling Pathway

In humans, the Hippo signaling pathway resides in a core kinase cascade comprising LATS1/2 and MST1/2 (Yamauchi and Moroishi, 2019[162]). LATS1/2 are phosphorylated and activated by MST1/2 in complex with the scaffold protein SAV1 and phosphorylate TAZ and YAP (Qi et al., 2022[119], Zhao et al., 2025[181]). However, the phosphorylation of YAP/TAZ leads it to bind 14-3-3 proteins and transport itself to the cytoplasm, forming a sequestering complex and ultimately marking it for proteasomal degradation (van Soldt and Cardoso, 2020[149]). The Hippo pathway maintains tight control of cell proliferation and apoptosis by preventing the formation of YAP/TAZ nuclear localization (Ehmer and Sage, 2016[32], Nie et al., 2020[107]). The dysregulation of this cascade through mutation, loss of upstream kinases, or inactivation of LATS1/2 results in accumulations of unphosphorylated YAP/TAZ in the nucleus that drives the transcription of gene sets related to cell development, survival, and metastasis (Messina et al., 2023[94]).

**Figure 2:**
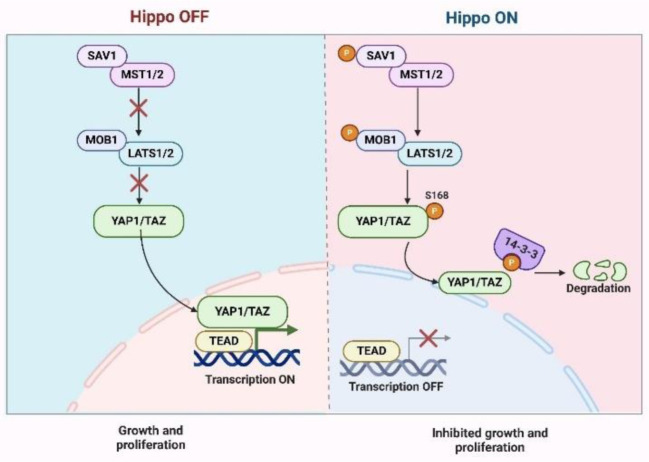
Figure 2 illustrates the Hippo signaling pathway in its "OFF" and "ON" states, highlighting its role in regulating cell growth and proliferation. In the Hippo OFF state, the kinase cascade involving MST1/2 and LATS1/2 is inactive, allowing YAP1/TAZ to translocate into the nucleus and associate with TEAD transcription factors, thereby promoting transcription of genes involved in growth and proliferation. Conversely, in the Hippo ON state, MST1/2, with the assistance of SAV1, activates LATS1/2 in a complex with MOB1. This leads to the phosphorylation of YAP1/TAZ, creating a binding site for the 14-3-3 protein, which sequesters YAP1/TAZ in the cytoplasm and promotes their degradation. As a result, YAP1/TAZ is prevented from entering the nucleus, inhibiting TEAD-mediated transcription and suppressing growth and proliferation. LATS1/2 - Large Tumor Suppressor Kinase 1 and 2; MOB1 - Mps One Binder Kinase Activator-like 1; MST1/2 - Mammalian Sterile 20-like Kinase 1 and 2; SAV1 - Salvador Homolog 1; TAZ - Transcriptional Coactivator with PDZ-binding Motif; TEAD - TEA Domain Family Member; YAP1 - Yes-associated Protein 1

The Hippo pathway is regulated by critical regulators that act upstream (Zhong et al., 2024[184]). The kinase cascade is regulated by mechanical signals (ECM stiffness and cellular tension), affecting actomyosin contractility (McKenzie et al., 2020[92]). YAP/TAZ activation is promoted by increased ECM stiffness, a characteristic of many cancers, allowing cells to adapt to their environment (Liang and Song, 2023[71]). Furthermore, cell polarity and junctional integrity, determined by angiomotin and E-Cad, respectively, regulate Hippo pathway activity via controlling YAP/TAZ localization (Ahmad et al., 2022[1]). The Hippo pathway also integrates with other signaling pathways to further integrate into broader cellular networks (Ibar and Irvine, 2020[54]). For example, YAP/TAZ cooperates with TGF-β on EMT and WNT signaling to induce tumorigenic outcomes (Savorani et al., 2021[130]). A second key modulator of Hippo pathway regulation is GPCR signaling, which either activates or inhibits YAP/TAZ in a receptor subtype and ligand type-dependent manner (Xu et al., 2020[156], Yu et al., 2012[169]). When dysregulated, this intricate network underscores the importance of the pathway as a tumor suppressor and a promotor of oncogenesis (Molinolo et al., 2009[100]). The hippo pathway controls YAP/TAZ nuclear activity, regulating cell growth and proliferation, as shown in Figure 2(Fig. 2).

## YAP/TAZ-Mediated Regulation of EMT and Metastasis

### Mechanisms of EMT induction

**Figure 3:**
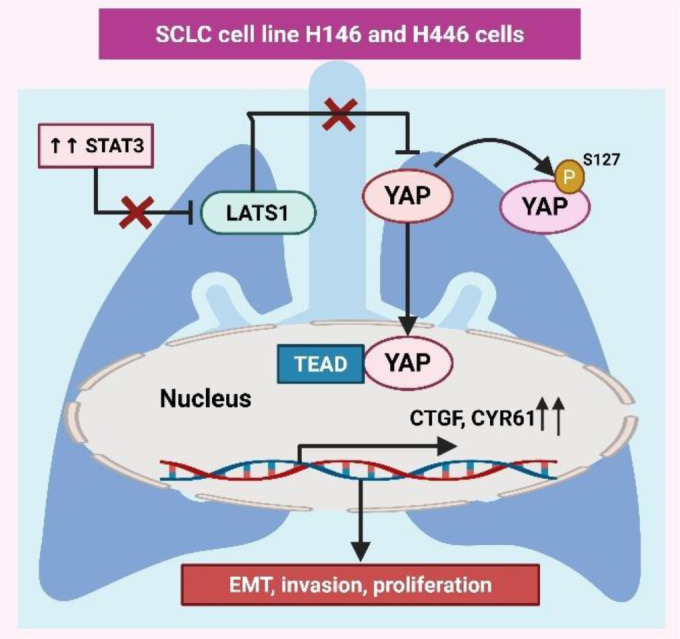
Figure 3 depicts the dysregulation of the Hippo signaling pathway in small cell lung cancer (SCLC) cell lines H146 and H446, showing the overexpression of STAT3, which inhibits LATS1 activity. This suppression of LATS1 prevents phosphorylation of YAP at S127, allowing unphosphorylated YAP to accumulate and translocate into the nucleus. In the nucleus, YAP is associated with TEAD transcription factors to activate the expression of target genes, including CTGF and CYR61, which promote EMT, invasion, and proliferation. CTGF - Connective Tissue Growth Factor; CYR61 - Cysteine-rich Angiogenic Inducer 61; EMT - Epithelial-Mesenchymal Transition; H146 - Human Small Cell Lung Cancer Cell Line H146; H446 - Human Small Cell Lung Cancer Cell Line H446; LATS1 - Large Tumor Suppressor Kinase 1; S127 - Serine 127; SCLC - Small Cell Lung Cancer; STAT3 - Signal Transducer and Activator of Transcription 3; TEAD - TEA Domain Transcription Factor; YAP - Yes-associated Protein

During EMT, epithelial cells are transformed into mesenchymal-like cells with increased migratory, invasive, and metastatic potential (Pedri et al., 2022[114]). Airway quiescence and inflammation are central to this process, and their execution depends on the major effectors of the Hippo signaling pathway, transcriptional coactivators TAZ and YAP (Lin et al., 2016[72], Zhang et al., 2018[177]). Activation of YAP/TAZ transcriptionally induces key transcription factors, Snail, ZEB1, ZEB2, and Twist, modulating transcriptional programs that promote cellular plasticity (Lopez-Hernandez et al., 2021[82]). Several factors suppress E-Cad, induce VIM and N-Cad, and allow cells to detach and migrate (Sulaiman et al., 2018[139]). Activations of YAP/TAZ are responded to by mechanical cues, cellular density, and ECM stiffness and are linked to TME conditions favorable for metastasis (Mierke, 2024[95], Tong et al., 2022[148]). YAP/TAZ binds to TEAD factors upon activation and induces EMT-related gene expression (Zhao et al., 2008[180]). Beyond that, YAP/TAZ integrates with pathways, including TGF-β and WNT/β-catenin, further augmenting their oncogenic potential (Park et al., 2015[112]). Specifically, TGF-β cooperates with YAP/TAZ at the SMAD complex to maintain EMT and promote tumor cell invasiveness (Ríos-López et al., 2023[123]). Cutting across multiple studies, YAP/TAZ is key in driving EMT (Sun et al., 2023[141], Yamaguchi and Taouk, 2020[161]). Hsu et al. demonstrated that STAT3 overexpression in small-cell lung cancer (SCLC) promotes EMT, proliferation, and invasion through YAP activation. STAT3 increased YAP expression, its downstream targets (CTGF, CYR61), and EMT markers (MMP-2, MMP-9), identifying the STAT3-YAP axis as a treatment target, as shown in figure 3(Fig. 3) (Hsu et al., 2022[48]). In pancreatic ductal adenocarcinoma (PDAC), GSK3β inhibition by 6-bromoindirubin-30-oxime (BIO) activated YAP by deactivating the hippo pathway, leading to nuclear localization (Li et al., 2016[68], Thongon et al., 2016[146]). YAP drives EMT in PDAC by regulating key markers like E-cadherin and vimentin, and its genetic ablation reduces cancer cell growth, underscoring its oncogenic role (Monkman et al., 2019[102]). Thongon et al. highlighted erlotinib and BIS I as modulators of YAP activity in PDAC, where BIS I suppresses YAP-dependent EMT and reduces migration, proliferation, and clonogenicity (Li et al., 2018[67], Thongon et al., 2016[146]). Similarly, ZEB1, a potent EMT activator, interacts with YAP in breast cancer to co-activate shared target genes (Feldker et al., 2020[33]). Lehmann et al. revealed this collaboration contributes to metastasis, therapy resistance, and poor survival, particularly in aggressive TNBC (Lehmann et al., 2016[66]). Feldker et al. identify ZEB1 as a key interactor of AP-1 factors (FOSL1, JUN) and YAP in TNBC, forming a transactivation complex that activates tumor-promoting genes while repressing epithelial genes. This mechanism drives aggression in claudin-low breast cancer (Feldker et al., 2020[33], Lou et al., 2023[83]). 

Environmental carcinogens have also been linked to YAP/TAZ-driven EMT (Zhou et al., 2023[185]). Gao et al. associated nitro-polycyclic aromatic hydrocarbons in PM2.5 with lung cancer metastasis, showing that these carcinogens inactivate MST1/2 and LATS1/2, leading to YAP nuclear translocation and transcription of pro-migration genes (Gao et al., 2019[36], Thapa et al., 2025[145]). Additionally, Xia et al. reported that high YAP expression correlates with poor survival in ovarian cancer, where it promotes EMT, migration, and drug resistance. At the same time, dominant-negative YAP mutants reverse these effects (Xia et al., 2014[154]). Lv et al. identified YAP1 activation as a driver of dedifferentiation and reprogramming of granulosa cells in high-grade serous ovarian cancer, highlighting its role in tumor initiation and progression (Lv et al., 2020[88], Sadique Hussain et al., 2025[127]). While these studies provide compelling evidence for the function of YAP/TAZ in EMT across various cancers, they also highlight key challenges. Many studies effectively validate the functional role of YAP/TAZ in vivo and in vitro. Still, there is a limited exploration of the heterogeneity in TME and its impact on YAP/TAZ activation. Additionally, most findings lack longitudinal data to confirm their translational relevance in clinical settings. Integrating YAP/TAZ with mechanical and biochemical signaling underscores their pivotal role in metastasis, but the exact mechanisms driving tumor-type-specific effects remain underexplored (Figure 3(Fig. 3)).

### Interaction with TGF-β signaling

YAP/TAZ and TGF-β signaling interplay is critical in promoting metastasis and EMT (Zhang et al., 2022[178]). TGF-β activates both SMAD-independent and SMAD-dependent pathways that synergize with YAP/TAZ to initiate tumor progression (Hussain et al., 2025[53], Miranda et al., 2017[96]). YAP/TAZ binds directly with SMAD proteins, forming transcriptional complexes that amplify the expression of EMT-related genes, including VIM and N-Cad ZEB1 and Snail (Cheng et al., 2020[18]). Beyond their transcriptional roles, YAP/TAZ modifies the TGF-β pathway by enhancing the stability and activation of TGF-β receptors, sustaining the signaling loop (Miranda et al., 2017[96]). This crosstalk drives EMT and facilitates tumor cell plasticity, enabling a hybrid epithelial/mesenchymal state that supports metastasis and therapy resistance (Coban et al., 2021[22]). 

Liu et al. demonstrated that YAP modulates cell fate by suppressing TGF-β1-induced apoptosis and promoting EMT in mouse mammary epithelial cells. Overexpression of YAP shifted the cellular response toward EMT, while YAP knockdown enhanced apoptosis and reduced EMT. This effect was mediated via EGFR activation, highlighting the dual role of YAP in determining cellular responses to TGF-β1 (Liu et al., 2017[76], Thapa et al., 2024[144]). Further downstream, the AXL receptor tyrosine kinase emerges as a critical effector of YAP-TEAD transcriptional activity (Xu et al., 2011[158]). AXL drives mitogenic and survival signals, promoting metastasis, invasion, and EMT while bypassing the anti-mitogenic effects of TKIs (Auyez et al., 2021[5]). In mesenchymal-type lung cancer, Choi et al. found that YAP and the TGFβ/SMAD axis were important regulators of AXL expression. The nuclear translocation of YAP was caused by doxorubicin treatment, and AXL expression was boosted by a combination of TGFβ/SMAD signaling and this process. Targeting this YAP-TGFβ/SMAD-AXL pathway holds promise for improving chemosensitivity in mesenchymal lung cancer (Choi et al., 2021[20], Thapa et al., 2024[143]). In melanoma, the transition from a melanocytic proliferator to a mesenchymal invader involves extensive transcriptional reprogramming driven by the interplay of multiple signaling pathways (Hossain and Eccles, 2023[47]). Lüönd et al. elucidated the hierarchical interaction between Wnt/β-catenin, YAP/TAZ, and TGFβ/SMAD signaling in driving this phenotype switch. SMAD-mediated transcription activated β-catenin and YAP/TAZ, with YAP/TAZ governing critical phenotype-switching programs. β-catenin further supported differentiation and phenotype switching but relied on SMAD and YAP/TAZ for activation. These findings clarify pathway convergence and suggest therapeutic priorities for melanoma (Jha et al., 2024[57], Lüönd et al., 2022[86]).

Environmental and dietary factors influence the YAP-TGF-β axis (Schmidt et al., 2024[132]). Deng et al. revealed that bisphenol S (BPS), an industrial chemical, promotes TNBC cell migration by activating YAP through LATS1 dephosphorylation and GPER signaling. This activation upregulated downstream targets such as CTGF and ANKRD1, enhancing mesenchymal markers like fibronectin and vimentin. Blocking GPER or YAP inhibited BPS-triggered migration, suggesting that the GPER/Hippo-YAP axis could be a treatment target for TNBC metastasis (Dahiya et al., 2024[24], Deng et al., 2018[27]). Another dietary compound, Resveratrol (RSVL), has demonstrated anti-cancer properties by modulating the Hippo-YAP pathway (Honari et al., 2019[46]). Deng et al. showed that RSVL inhibits EMT in gastric cancer cells produced by TGF-β1 via suppressing YAP activity, reducing migration, invasion, and tumor growth in vivo. These findings highlight RSVL as a promising therapeutic agent targeting the Hippo-YAP axis in gastric cancer (Deng et al., 2022[26]). These studies collectively underscore the complexity and significance of the YAP/TAZ-TGF-β signaling axis in promoting EMT and metastasis. While they provide convincing evidence of the cooperative role of YAP/TAZ and TGF-β, several limitations remain. Many findings rely on in vitro and murine models, with limited validation in human clinical settings (Sharma et al., 2023[134]). Additionally, the heterogeneity of tumor microenvironments and their influence on pathway dynamics are underexplored. Future research should focus on uncovering the spatial and temporal dynamics of YAP/TAZ and TGF-β interactions in vivo. Therapeutically, targeting downstream effectors such as AXL or modulating the YAP-TGF-β signaling axis with agents like resveratrol offers promising avenues for managing EMT-driven metastasis.

### YAP/TAZ as promising cancer biomarkers

The dysregulation of YAP/TAZ in several cancers characterizes them as a hallmark for diagnosis, prognosis prediction, and therapeutic response. High levels of nuclear YAP/TAZ expression are associated with aggressive tumor phenotypes, therapy resistance, and poor clinical outcomes in various cancers, including lung, breast, ovarian, liver, and renal cancer (Gupta et al., 2023[40], Yang et al., 2024[164]). To take an example, in non-small cell lung cancer (NSCLC), the high YAP1 levels are related to the resistance to EGFR-TKI and metastasis. In contrast, in triple-negative breast cancer (TNBC), YAP/TAZ activation promotes cancer stemness and chemoresistance and suggests prediction for disease progress. Moreover, YAP/TAZ interact with significant signaling pathways (e.g., TGF-β. Wnt) and modify EMT markers, which also endorse their possibility of a biomarker for detection (Cheng et al., 2024[19]). Their identification in tumor micro-environment or LIQUID BIOPSI could help with early diagnosis, patient selection, and treatment efficacy monitoring. Yet, challenges persist, including deviations in the detection methods standardization, heterogeneity of YAP/TAZ expression within tumor cells, and distincton their tumor-promoting roles and their physiological functions (Prasher et al., 2022[118], Zhang et al., 2024[176]). However, gathering sufficient protein sources to accurately establish the arsenal of tools that exist for screening YAP/TAZ and other protein serology candidates is daunting, but combining YAP/TAZ quantification with additional molecular markers might enhance the precision of oncology treatments, directing therapies onto targets and the goal of having an improved patient outcome (Bayraktar et al., 2023[6]). Future studies should validate their efficiency in large clinical cohorts and develop orthogonal detection platforms.

## Roles of YAP/TAZ in EMT and Cancer Progression

### Lung cancer

The most prevalent contributor to cancer fatalities globally is lung cancer, with NSCLC representing 80 % of cases (Molina et al., 2008[99]). Early metastasis in NSCLC reduces the survival rate to under 15 % after 5 years (Zappa and Mousa, 2016[173]). EMT, driven by factors such as TGF-β and tyrosine kinase receptors (e.g., IGF and PDGF), plays a pivotal role in metastasis by repressing epithelial markers like E-Cad and activating mesenchymal transcription factors, including SNAI1, Slug, ZEB1, and Twist (Savagner, 2010[129]). The Hippo signaling pathway acts as a cancer suppressor, regulating YAP1, a key driver of tumor growth, metastasis, and stem cell properties (Zhou et al., 2023[185]). While Hippo pathway activation restricts YAP1 activity, its inactivation leads to YAP1-mediated tumor progression and poor patient outcomes (Alharbi et al., 2022[2], Mohajan et al., 2021[97]). Yu et al. identified YAP1 as a critical driver of NSCLC metastasis through EMT induction. YAP1 activates Slug transcription via YAP1/TEAD interaction, enhancing proliferation, migration, invasion, and EMT marker expression. Verteporfin, a YAP1 inhibitor, disrupts YAP1/TEAD-mediated EMT, highlighting YAP1 as a promising treatment target for NSCLC (Yu et al., 2018[171]). Resistance to EGFR-TKIs remains a significant challenge in NSCLC, driven by both primary and acquired resistance mechanisms (Morgillo et al., 2016[104]). Lee et al. demonstrated that YAP activation contributes to EGFR-TKI resistance by inducing AXL and ERK signaling, independent of the Hippo pathway. Combining YAP inhibition with EGFR-TKI therapy overcame resistance in lung adenocarcinoma models, suggesting a novel treatment strategy (Lee et al., 2016[63]). Angiomotin (AMOT), a scaffold protein, has become a critical controller of YAP/TAZ activity in lung cancer (Moon et al., 2018[103]). Hsu et al. identified AMOT as a tumor suppressor, demonstrating that AMOT loss promotes EMT, invasion, migration, and proliferation by reducing the cytoplasmic sequestration of YAP/TAZ and increasing their nuclear localization. This shift upregulates Cyr61, enhancing metastatic capacity. AMOT knockdown accelerates lung cancer metastasis in vivo and in vitro, underscoring its role as a prognostic biomarker and a possible lung cancer treatment target (Hsu et al., 2015[49]). Verteporfin (VP), an FDA-approved drug, inhibits YAP-TEAD communication. At the same time, WWC3, a member of the WWC gene family, activates the Hippo pathway by interacting with LATS, thereby suppressing EMT (Wei and Li, 2020[153]). Han et al. identified WWC3 as a tumor suppressor in NSCLC, regulating YAP/LATS1 phosphorylation to reduce mesenchymal marker expression and invasiveness. WWC3 knockdown enhanced EMT and tumor aggressiveness, while its overexpression suppressed tumorigenic traits, promising as targets for lung cancer therapy (Han et al., 2018[41]).

YAP/TAZ signaling interacts with lncRNAs and miRNAs to control tumor progression (Zhang et al., 2022[178]). Sardo et al. implicated YAP/TAZ in NSCLC progression through miR-106b-25 cluster-mediated suppression of genes that inhibit tumor growth, like TGFBR2. YAP/TAZ cooperates with EZH2 to modulate miRNA and lncRNA networks, driving oncogenesis and therapy resistance. This cooperation highlights EZH2 and YAP/TAZ as possible treatment targets in NSCLC (Lo Sardo et al., 2021[78]). PTEN, a key tumor suppressor, is frequently downregulated in NSCLC, leading to hyperactivation of the PI3K/AKT/mTOR pathway and promoting progression, stemness, and therapy resistance (Luongo et al., 2019[87]). Sardo et al. demonstrated that YAP/TAZ, in collaboration with EZH2 and MYC, represses PTEN transcriptionally, maintaining its low levels in lung adenocarcinoma (LUAD). This axis correlates with poor prognosis, and targeting YAP/TAZ-EZH2-MYC restored PTEN expression and reduced tumor growth, suggesting a novel therapeutic avenue (Lo Sardo et al., 2024[80]). Emerging evidence underscores the role of lncRNAs in YAP/TAZ-driven tumor progression (Zhao et al., 2023[183]). Zhu et al. identified SFTA1P as a YAP/TAZ-regulated lncRNA that enhances YAP/TAZ activity by stabilizing TAZ mRNA, promoting proliferation and EMT. Its knockdown inhibited tumorigenic potential, making it a promising treatment target in NSCLC (Zhu et al., 2021[186]). Similarly, CD109, an oncogene in lung adenocarcinoma, enhances EMT traits and stemness by activating YAP/TAZ, further driving metastasis. Lee et al. identified CD109 as a prognostic biomarker and therapeutic target (Lee et al., 2020[64]).

Therapeutic strategies targeting YAP/TAZ have shown promise in overcoming therapy resistance and suppressing EMT (Nguyen and Yi, 2019[105]). Xu et al. demonstrated that a YAP/TAZ inhibitor reduced EGFR-TKI resistance in NSCLC with EGFR mutations (L858R/T790M) by downregulating YAP/TAZ activity and inhibiting the ERK1/2 pathway. This approach sensitized resistant cells to EGFR-TKIs and induced apoptosis, highlighting its potential as a treatment candidate (Xu et al., 2019[157]). Emerging natural compounds, such as corosolic acid (CA) and cytochalasin H (CyH), exhibit anti-tumor activity by targeting the YAP/TAZ pathway. CA inhibits EMT and metastasis in NSCLC by suppressing YAP-mediated gene expression and initiating ferroptosis (Zhang et al., 2024[175]). Similarly, CyH disrupts YAP-TEAD interaction and suppresses EMT and cancer stemness, making it another promising therapeutic agent for YAP/TAZ-driven cancers (Xiu et al., 2021[155]). These studies emphasize the critical role of YAP/TAZ in NSCLC therapy resistance, progression, and EMT. While significant strides have been made, challenges remain in translating these findings into clinical applications. Future efforts should focus on developing selective YAP/TAZ inhibitors and combinatorial therapies that target their interactions with key regulators like EZH2 and TEAD. Such approaches can potentially enhance results in YAP/TAZ-driven lung cancers.

### Breast cancer

One of the leading causes of cancer-related fatalities globally is breast cancer, which is also the most common disease among women (Arnold et al., 2022[4]). Aberrant expression of miRNAs, key post-transcriptional regulators, significantly contributes to breast cancer development and progression (Loh et al., 2019[81]). Moreover, advanced breast cancers frequently exhibit dysregulation of the Hippo pathway, which influences tumor progression, metastasis, and therapy resistance (Sadri et al., 2024[128]). Canu et al. demonstrated that SPAG5, vital for mitotic spindle function, is an immediate target for transcription of YAP/TAZ/TEAD in breast cancer. Elevated SPAG5 protein levels correlate with poor disease-free survival and aggressive tumor behavior. The YAP/TAZ-driven feedback loop, mediated by miR-10b-3p, amplifies SPAG5 expression, highlighting YAP/TAZ or SPAG5 as potential therapeutic targets in breast cancer (Canu et al., 2021[11]). YAP activity, characterized by nuclear translocation and TEAD interaction, is a major contributor to TNBC aggressiveness (Luo et al., 2023[85]). Parambil et al. identified YAP as a key driver of TNBC progression via activation of the EGFR-AKT axis. YAP enhances proliferation, migration, and survival while preventing apoptosis. RNA interference or pharmacological inhibition of YAP significantly reduces these effects, underscoring its promise as a target for treatment in TNBC, especially in xenograft models generated from patients (Parambil et al., 2023[111]). In TNBC, YAP and TAZ are crucial for maintaining CSC self-renewal and tumor initiation, correlating with aggressive histology and metastasis (Fultang et al., 2021[35]). Vici et al. reported that combined expression of YAP/TAZ in tumor and stromal cells predicts lower pCR rates and reduced DFS. These findings position YAP/TAZ as prognostic markers and therapeutic targets in TNBC management (Vici et al., 2016[151]). The role of YAP/TAZ in therapy resistance is further exemplified in HER2-positive breast cancer (Zhao et al., 2023[183]). Alonso et al. identified TEAD2 overexpression and YAP1 dephosphorylation as key contributors to trastuzumab resistance. Blocking YAP1/TEAD complexes restored trastuzumab sensitivity, suggesting that dual targeting of HER2 and YAP1/TEAD could improve outcomes in HER2-positive breast cancer individuals (González-Alonso et al., 2020[38]).

Natural compounds, such as luteolin and apigenin, have shown promising anti-cancer effects by targeting the YAP/TAZ pathway (Singh Tuli et al., 2022[138]). Cao et al. revealed that luteolin promotes YAP/TAZ degradation, suppressing EMT in TNBC. Luteolin reduces mesenchymal markers, enhances epithelial markers, and inhibits migration and tumor growth in vivo, making it a possible medicinal agent for TNBC (Cao et al., 2020[12]). Similarly, Li et al. demonstrated that apigenin disrupts YAP/TAZ-TEAD interaction and downregulates CYR61 and CTGF genes, reducing proliferation, migration, and stemness in TNBC cells. These findings highlight apigenin's therapeutic potential for YAP/TAZ-driven breast cancer (Li et al., 2018[69]). Beyond natural compounds, metformin, a widely used antidiabetic drug, has garnered attention for inhibiting EMT and YAP/TAZ activity in breast cancer (Amengual-Cladera et al., 2024[3]). Xu et al. reported that metformin suppresses YAP expression and EMT regulation, reducing chemoresistance and metastasis in HER2-positive and TNBC patients. These findings suggest metformin as a low-cost, well-tolerated YAP/TAZ inhibitor with significant therapeutic potential (Xu et al., 2023[160]). Estrogen receptor α36 (ERα36), a splice variant of ERα, is linked to tamoxifen resistance and TNBC development (Maczis et al., 2018[89]). Park et al. revealed that ERα36 enhances YAP activity via Src kinase, contributing to tamoxifen resistance and aggressive breast cancer phenotypes. YAP knockout reversed these effects, highlighting the potential of YAP targeting in ERα36-overexpressing breast cancers (Park et al., 2022[113]). These findings collectively underscore the pivotal role of YAP/TAZ in breast tumor development, metastasis, and resistance to therapy. Targeting the YAP/TAZ pathway with pharmacological inhibitors, natural compounds, or combination therapies offers hope for better results in groups of aggressive breast cancer.

### Ovarian cancer

The deadliest type of ovarian cancer is epithelial ovarian cancer (EOC), which is the third most common type of cancer in women (Desai et al., 2014[28]). Its poor prognosis stems from late-stage diagnosis, extensive metastasis, and high rates of chemoresistance (Ramos et al., 2021[121]). Dysregulation of key signaling pathways, such as PI3K/AKT/mTOR, MAPK, and the Hippo pathway, contributes significantly to ovarian cancer growth and development (Rascio et al., 2021[122]). The Hippo pathway regulates YAP/TAZ activity, and its dysregulation, resulting in nuclear YAP expression, has been linked to tumorigenesis and poor survival, highlighting its therapeutic potential (Calses et al., 2019[10]). Chen et al. demonstrated that TAZ exaggeration drives ovarian cancer progression, promoting EMT, migration, and proliferation. High TAZ levels correlate with poor patient outcomes, and TAZ knockdown or YAP/TAZ-TEAD inhibition reduces EMT markers, supporting the potential of TAZ as a therapeutic target (Chen et al., 2016[15]). The Wnt pathway, particularly its non-canonical β-catenin-independent branch, is essential in ovarian cancer (Nguyen et al., 2019[106]). Ghobadi et al. showed that Wnt5A mediates EMT through TGF-β1/Smad2/3 and Hippo-YAP/TAZ crosstalk, driving YAP nuclear translocation and enhancing invasion. Verteporfin, a YAP1 inhibitor, decreases Wnt5A expression and EMT markers, emphasizing the potential of targeting Wnt5A in ovarian cancer (Dehghani-Ghobadi et al., 2022[25]). Endothelin-1 (ET-1) signaling has emerged as another critical driver of ovarian cancer progression (Tocci et al., 2021[147]). Sestito et al. identified the ET-1/ETAR axis as a promoter of EMT and metastasis in HG-SOC. ET-1 enhances YAP/ZEB1 nuclear interaction, forming a transcriptional complex with AP-1 to sustain tumor progression. ETAR blockade with macitentan suppresses metastasis in vivo, with high ETAR/ILK/YAP/ZEB1 expression predicting poor prognosis (Sestito et al., 2022[133]). Amphiregulin (AREG), an EGFR ligand, is overexpressed in several cancers, including ovarian cancer, where it contributes to cancer development and metastasis (Bolitho et al., 2021[8]). Jia et al. demonstrated that AREG promotes invasion in epithelial ovarian cancer by activating YAP. AREG downregulates E-cadherin while upregulating Egr-1 and Slug, with YAP as an essential mediator. The AREG/YAP-induced signaling axis correlates with poor survival, highlighting its therapeutic potential (Jia et al., 2024[58]). 

Honokiol (HNK), a bioactive compound derived from Magnolia species, has demonstrated potent anticancer effects in ovarian cancer (Ong et al., 2019[108]). Liu et al. revealed that HNK suppresses EMT, invasion, and migration by downregulating the YAP/TAZ pathway. HNK-induced apoptosis and tumor growth inhibition in vivo were reversed by the YAP agonist XMU-MP-1, underscoring the centrality of the YAP/TAZ pathway in ovarian cancer progression and HNK's therapeutic potential (Liu et al., 2024[74]). HGSC subtype is often characterized by malignant ascites, metastasis, and chemoresistance (Lisio et al., 2019[73]). Pietilä et al. reported that platinum-based chemotherapy alters the extracellular matrix (ECM) composition, promoting resistance via FAK, β1 integrin-pMLC-YAP signaling. Upregulated COL6 enhances ECM stiffness and adhesion signaling, driving apoptosis resistance and tumor recurrence. Targeting ECM components could provide new strategies to combat metastasis and therapy resistance in HGSC (Pietilä et al., 2021[116]). These findings collectively underscore the critical role of YAP/TAZ and associated pathways in ovarian cancer metastasis, progression, and chemoresistance. The therapeutic potential of targeting YAP/TAZ and related signaling molecules such as Wnt5A, ET-1, AREG, and ECM components offers promising avenues for improving outcomes in ovarian cancer patients.

### Liver cancer

The molecular and clinical heterogeneity of liver cancer, along with poorly understood mechanisms of progression, complicates therapeutic strategies (Foglia et al., 2023[34]). Genetic alterations in cell growth and migration pathways, including Hippo signaling dysregulation and EMT, are central to HCC progression (van Zijl et al., 2009[150]). The loss of liver kinase B1 (LKB1) has been implicated in HCC progression (Geng et al., 2022[37]). Qiu et al. demonstrated that LKB1 loss induces EMT via ZEB1 upregulation, which regulates YAP expression. Elevated YAP activity drives downstream gene activation, promoting motility, invasiveness, and malignant progression. LKB1 overexpression reverses these effects, highlighting the ZEB1-YAP axis as a therapeutic target (Qiu et al., 2018[120]). TAZ is upregulated in HCC and regulated by the TGF-β/SMAD signaling axis (Choi and Kim, 2024[21]). According to López et al., TGF-β causes TAZ expression in HepG2 cells via the classical SMAD route, linking TGF-β and Hippo pathway crosstalk. TAZ overexpression contributes to HCC progression, making it a possible target for therapy (Ríos-López et al., 2023[124]). Targeting YAP/TAZ to enhance the efficacy of existing therapies has shown promise (Kumar et al., 2024[62]). Han et al. demonstrated that combining a YAP inhibitor (CA3) with sorafenib, the standard first-line treatment for HCC, is particularly effective in high YAP/TAZ-expressing tumors. The combination improves sensitivity to sorafenib, offering a novel therapeutic strategy (Han et al., 2022[42]). The TME in HCC is characterized by hypoxia, which promotes angiogenesis, metabolic reprogramming, and EMT (Chen et al., 2022[14]). Liu et al. revealed that hypoxia-conditioned mesenchymal stem cells (hypo-MSCs) enhance HCC progression via the COX2/PGE2/EP4 axis, activating YAP and driving proliferation through the AKT/mTOR/SREBP1 pathway. Targeting EP4 or YAP under hypoxic conditions could mitigate tumor growth and improve therapeutic outcomes (Liu et al., 2019[77]). Biophysical factors in the tumor microenvironment, such as fluid shear stress (FSS), also contribute to HCC metastasis (Huang et al., 2018[52]). Yu et al. showed that FSS induces YAP nuclear translocation by disrupting cytomembrane binding with integrin β and upregulating GEF-H1, driving cytoskeletal rearrangement. Nuclear YAP activates EMT-related genes, such as SNAI1, enhancing motility and invasiveness. This FSS-YAP axis presents a possible treatment target (Yu et al., 2021[170]).

The role of PDCD10 in promoting HCC progression highlights another avenue for intervention (Liu et al., 2022[75]). Sun et al. demonstrated that PDCD10 enhances EMT and metastasis via PP2Ac-mediated YAP activation. High PDCD10 levels correlate with poor prognosis, and targeting PP2Ac with inhibitors like LB100 effectively suppresses tumor growth and metastasis, supporting PDCD10 as a potential target for therapy (Sun et al., 2021[140]). Amphiregulin (AREG), frequently overexpressed in HCC, has also been identified as a biomarker and driver of tumor progression (Isaac et al., 2021[56]). Han et al. demonstrated that AREG levels correlate with the Edmondson stage and prognosis. AREG exceeds AFP levels in sensitivity as a serum biomarker and is closely associated with YAP and TAZ activity, further emphasizing its diagnostic and therapeutic significance in HCC (Han et al., 2014[43]). These findings highlight the critical role of Hippo pathway dysregulation and associated signaling mechanisms in HCC progression and metastasis. The development of combination therapies targeting YAP/TAZ, alongside existing treatments like sorafenib, and strategies addressing the TME, such as hypoxia and FSS, offers promising therapeutic potential.

### Renal cancer

Renal cell carcinoma (RCC) accounts for 2-5 % of adult malignancies, with clear cell RCC (ccRCC) being the most prevalent and aggressive subtype, comprising 75-85 % of cases (Yang et al., 2023[165]). Often asymptomatic in its early stages, ccRCC is frequently diagnosed at advanced stages with metastasis, leading to high mortality rates (Schiavoni et al., 2023[131]). Despite therapeutic advancements, RCC remains incurable mainly, emphasizing the need for further investigation into its molecular mechanisms (McKay et al., 2018[91]). Angiogenesis is a hallmark of RCC, driven by mutations in the VHL gene that lead to excessive activation of HIFs and increased production of angiogenic factors such as VEGF and PDGF (Chappell et al., 2019[13]). In addition to angiogenesis, the TME contributes much to RCC progression (Heidegger et al., 2019[44]). Chen et al. identified YAP1 as a mechanosensor meditating low-shear stress-induced EMT and metastasis in RCC. YAP1 activation under low shear stress enhances nuclear localization, downregulates p-YAP1, and increases EMT markers such as N-Cad, SNAIL1, and Twists. Salvianolic acid B inhibits YAP1, reversing EMT and promoting apoptosis, highlighting its therapeutic potential (Chen et al., 2022[17]). TAZ is frequently elevated in RCC and correlates with poor prognosis, high Fuhrman grade, and metastasis (Mondal et al., 2024[101]). Ruan et al. demonstrated that TAZ is an independent prognostic marker, with its knockdown reducing cancer development in vivo and in vitro. This positions TAZ as a promising RCC diagnostic, predictive, and therapeutic target (Ruan et al., 2019[125]). SATB2, a nuclear matrix protein, plays an oncogenic role in RCC by coordinating chromatin remodeling (Chen and Costa, 2018[16]). Jin et al. found that YAP/TEAD4 activates SATB2, enhancing RCC proliferation and self-renewal. SATB2 inhibition sensitizes RCC to chemotherapy and suppresses YAP-high tumors in patient-derived models, highlighting its therapeutic potential (Jin et al., 2023[59]). Microphthalmia-associated transcription factor (MITF), a bHLH-LZ transcription factor, also contributes to ccRCC progression (Shibahara et al., 2001[135]). Kim et al. demonstrated that MITF activates the RhoA/YAP signaling pathway, promoting proliferation, migration, and invasion. MITF knockdown reduces tumor growth and metastatic potential, identifying MITF as a promising treatment target in ccRCC (Kim et al., 2021[61]).

**Figure 4:**
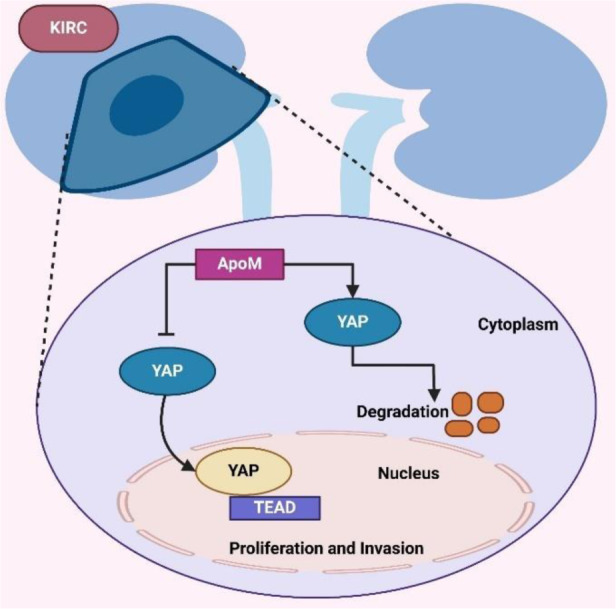
Figure 4 illustrates the role of ApoM in regulating YAP activity in kidney renal clear cell carcinoma (KIRC). ApoM inhibits the degradation of YAP in the cytoplasm, allowing YAP to accumulate and translocate into the nucleus. YAP interacts with TEAD transcription factors in the nucleus to drive the expression of genes that promote proliferation and invasion. ApoM - Apolipoprotein M; KIRC - Kidney Renal Clear Cell Carcinoma; TEAD - TEA Domain Transcription Factor; YAP - Yes-associated Protein

Mechanical signals within the ECM also play a critical role in RCC progression (Popova and Jücker, 2022[117]). PIEZO1, a mechanosensitive ion channel, mediates the effects of matrix stiffness on ccRCC via the Ca2+/Calpain/YAP pathway (Zhu et al., 2024[187]). Zhu et al. showed that PIEZO1 activation promotes proliferation, EMT, and stemness, while PIEZO1 deficiency disrupts these processes, reducing YAP nuclear translocation and tumor progression. PIEZO1 is thus a potential target for ccRCC treatment (Zhu et al., 2025[188]). Apolipoprotein M (ApoM), involved in lipid transport, has also been implicated in ccRCC (Borup et al., 2015[9]). Xu et al. found that ApoM levels are reduced in ccRCC tissues and linked to an undesirable prognosis. ApoM overexpression inhibits proliferation, EMT, and metastasis by attenuating Hippo-YAP protein expression and YAP stability, as shown in Figure 4(Fig. 4), making ApoM a possible target for ccRCC treatment (Xu et al., 2023[159]). Due to their unique physicochemical properties, zinc oxide nanoparticles (ZnO NPs) are emerging as novel anticancer agents (Bisht and Rayamajhi, 2016[7]). Wang et al. demonstrated that ZnO NPs promote ferroptosis in RCC cells by aiming the miR-27a-3p/YAP axis. ZnO NPs downregulate YAP expression, repress GPX4 and SLC7A11, and increase reactive oxygen species (ROS) and iron levels, inhibiting RCC invasion, migration, and proliferation in vivo and in vitro. These findings position ZnO NPs as potential therapeutic agents for RCC (Wang et al., 2022[152]). These studies emphasize the central role of Hippo pathway dysregulation and mechanosensitive signaling in RCC progression, metastasis, and therapy resistance. Targeting key players such as YAP1, TAZ, PIEZO1, and ApoM, as well as leveraging innovative agents like ZnO NPs, offers promising therapeutic strategies for RCC. Future research should focus on translating these findings into clinical applications and developing combination therapies to improve outcomes in advanced RCC (Figure 4(Fig. 4); Table 1(Tab. 1); References in Table 1: Canu et al., 2021[11]; Cao et al., 2020[12]; Chen et al., 2016[15], 2022[17]; Dehghani-Ghobadi et al., 2022[25]; González-Alonso et al., 2020[38]; Han et al., 2014[43], 2018[41], 2022[42]; Hsu et al., 2015[49]; Jia et al., 2024[58]; Jin et al., 2023[59]; Kim et al., 2021[61]; Lee et al., 2016[63], 2020[64]; Li et al., 2018[69]; Liu et al., 2019[77], 2024[74]; Lo Sardo et al., 2021[78], 2024[80]; Parambil et al., 2023[111]; Park et al., 2022[113]; Pietilä et al., 2021[116]; Qiu et al., 2018[120]; Ríos-López et al., 2023[124]; Ruan et al., 2019[125]; Sestito et al., 2022[133]; Sun et al., 2021[140]; Vici et al., 2016[151]; Wang et al., 2022[152]; Xiu et al., 2021[155]; Xu et al., 2019[157], 2023[159], 2023[160]; Yu et al., 2018[171], 2021[170]; Zhang et al., 2024[175]; Zhu et al., 2021[186]).

## Limitations of YAP/TAZ Inhibitors and Therapeutic Strategies

YAP/TAZ inhibitors, alongside various other therapeutic approaches, encounter several obstacles that prevent their translation into clinical settings. The dual functionality of YAP/TAZ between cancer promotion and tissue maintenance creates challenges because general inhibition could harm normal tissue homeostasis, including organ repair and wound healing processes (Huang et al., 2022[50]). YAP/TAZ activation shows varied patterns between distinct cancer types and unique tumors, which makes it difficult to create broadly applicable inhibitors because precise patient classification is required to prevent ineffective or dangerous results (Zhu et al., 2024[189]). The connection between YAP/TAZ proteins and other cancer-promoting signaling pathways, such as TGF-β and Wnt, usually generates backup survival mechanisms and treatment resistance that hinders ongoing treatments. Current research faces a major setback because developing potent and selective small-molecule inhibitors for YAP/TAZ-TEAD interactions faces two main drawbacks: these compounds frequently show undesirable secondary effects and unfavorable pharmacokinetic behavior (Yang et al., 2024[163]). Natural compounds, while less toxic, face issues of low bioavailability and insufficient preclinical validation. The temporal modifications within the tumor microenvironment, mechanical stimuli, and stromal interactions might reduce the effectiveness of YAP/TAZ-targeted treatment strategies in living organisms. The successful translation of preclinical discoveries into clinical benefits will depend on developing combination treatments because they address toxicities while advancing biomarker applications and better delivery methods (Yang et al., 2024[166]).

## Conclusion and Future Perspectives

As key players in the Hippo signaling system, YAP and TAZ promote resistance to treatment, metastasis, tumor aggressiveness, and EMT in a variety of cancer types. Through their oncogenic potential, this review has explored the role of YAP/TAZ as molecular hubs that integrate mechanical, biochemical, and metabolic cues from the TME. Combined, they drive their impact on tumor progression and resistance mechanisms, with TGF-β, WNT, and PI3K/AKT pathways significantly amplifying their effect. YPCT's role in EMT goes beyond transcriptional regulation to modify the TME and immune evasion. For example, fluid shear stress and extracellular matrix stiffness have been shown to activate YAP, facilitating cytoskeletal rearrangements and promoting invasion. In ovarian and renal cell carcinoma cancers, YAP/TAZ interacts with mechanosensitive ion channels like PIEZO1 and signaling molecules like AREG, driving tumor proliferation, migration, and stemness. The TAZ-YAP axis also creates chemoresistant phenotypes, as seen in platinum-resistant ovarian carcinoma and EGFR-TKI-resistant lung cancer.

Furthermore, YAP and TAZ impact the metabolic landscape of tumors observed in hepatocellular carcinoma, where they control lipogenesis by hypoxia-induced mesenchymal stem cells. Small molecule inhibitors, natural compounds, luteolin, apigenin, honokiol, and other emerging therapeutic strategies have been developed to target YAP/TAZ, including novel agents ZnONPs. However, combination therapies, such as YAP inhibitors with sorafenib in liver cancer or trastuzumab in breast cancer, have been promising in overcoming therapy resistance. In addition to their role in tumor cells, YAP/TAZ are central regulators of stromal elements, including CAFs, that sustain tumor growth and escape immune surveillance. In addition, therapeutic targeting of these interactions could improve treatment efficacy further. However, despite significant progress, these spatiotemporal dynamics of YAP/TAZ signaling remain incomplete in heterogeneous tumor microenvironments. The dual role of oncogenic drivers and modulators of immune and stromal responses requires a nuanced therapeutic approach. One major gap remains in identifying biomarkers for patient stratification to ensure targeted therapies are matched to molecular and pathological contexts that dictate response.

## Declaration

### Funding 

This work received no external funding. 

### Conflict of interest

The authors declare that no commercial or financial relationship during this study could be construed as a potential conflict of interest.

### Artificial intelligence usage 

The authors declare that no artificial intelligence tool was used throughout the conduction of this review. All research processes were completely performed by humans and all provided information are humanly generated.

## Figures and Tables

**Table 1:**
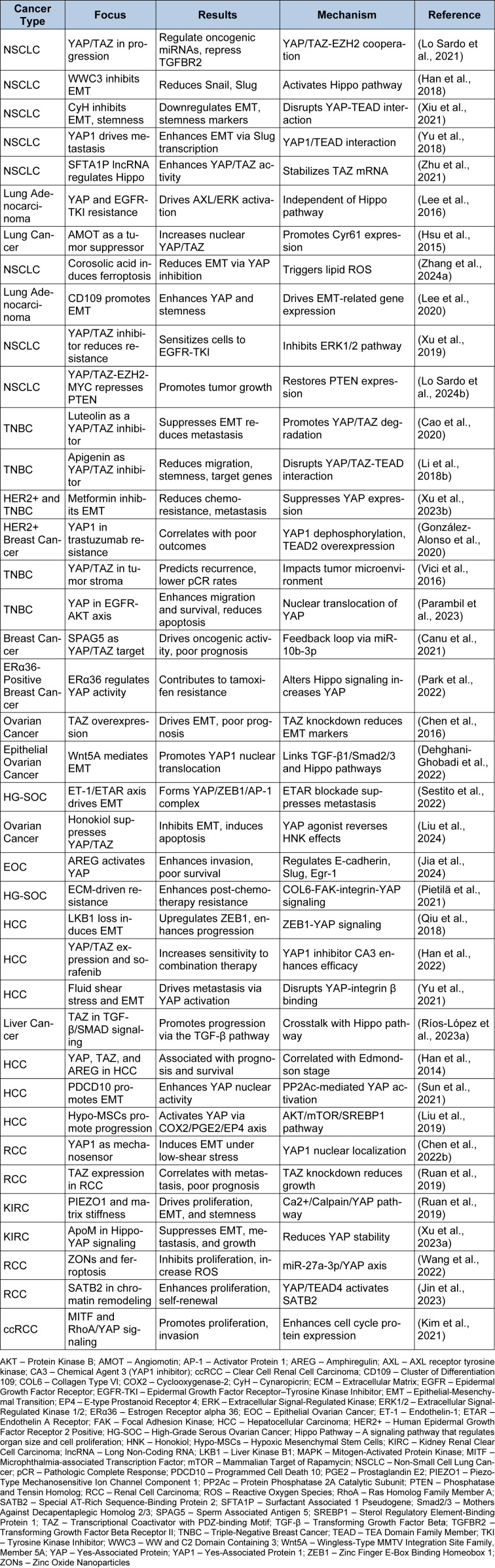
Summary of studies linking YAP/TAZ signaling to cancer progression and therapy.

**Figure 1:**
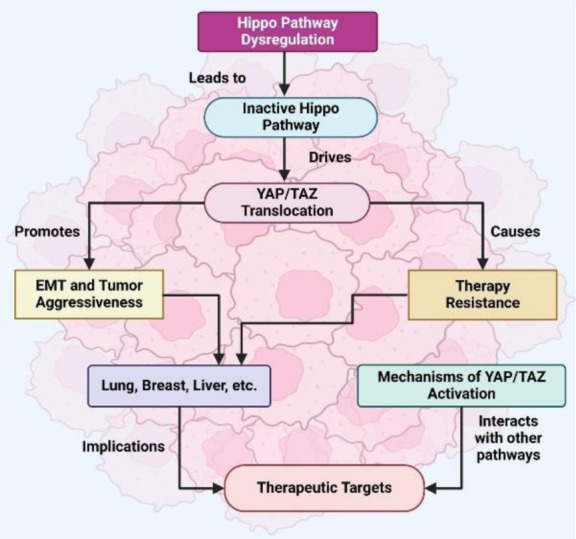
Graphical abstract
